# Evaluating the Soil Quality Index Using Three Methods to Assess Soil Fertility

**DOI:** 10.3390/s24030864

**Published:** 2024-01-29

**Authors:** Hiba Chaudhry, Hiteshkumar Bhogilal Vasava, Songchao Chen, Daniel Saurette, Anshu Beri, Adam Gillespie, Asim Biswas

**Affiliations:** 1 School of Environmental Sciences, University of Guelph, Guelph, ON N1G 2W1, Canada; hchaud02@uoguelph.ca (H.C.); hvasava@uoguelph.ca (H.B.V.); dsaurett@uoguelph.ca (D.S.); agilles@uoguelph.ca (A.G.); 2 ZJU-Hangzhou Global Scientific and Technological Innovation Center, Xiaoshan District, Hangzhou 311215, China; chensongchao@zju.edu.cn

**Keywords:** soil quality index, spectroscopy, fertility analysis, soil health indicators

## Abstract

Soil health plays a crucial role in crop production, both in terms of quality and quantity, highlighting the importance of effective methods for preserving soil quality to ensure global food security. Soil quality indices (SQIs) have been widely utilized as comprehensive measures of soil function by integrating multiple physical, chemical, and biological soil properties. Traditional SQI analysis involves laborious and costly laboratory analyses, which limits its practicality. To overcome this limitation, our study explores the use of visible near-infrared (vis-NIR) spectroscopy as a rapid and non-destructive alternative for predicting soil properties and SQIs. This study specifically focused on seven soil indicators that contribute to soil fertility, including pH, organic matter (OM), potassium (K), calcium (Ca), magnesium (Mg), available phosphorous (P), and total nitrogen (TN). These properties play key roles in nutrient availability, pH regulation, and soil structure, influencing soil fertility and overall soil health. By utilizing vis-NIR spectroscopy, we were able to accurately predict the soil indicators with good accuracy using the Cubist model (R^2^ = 0.35–0.93), offering a cost-effective and environmentally friendly alternative to traditional laboratory analyses. Using the seven soil indicators, we looked at three different approaches for calculating and predicting the SQI, including: (1) measured SQI (SQI_m), which is derived from laboratory-measured soil properties; (2) predicted SQI (SQI_p), which is calculated using predicted soil properties from spectral data; and (3) direct prediction of SQI (SQI_dp), The findings demonstrated that SQI_dp exhibited a higher accuracy (R^2^ = 0.90) in predicting soil quality compared to SQI_p (R^2^ = 0.23).

## 1. Introduction

Achieving a more productive agri-food sector is a current challenge. According to Saiz-Rubio et al. [1], we will need to increase global food production by 60% by the year 2050 due to the increasing population growth of over nine billion. Soil health plays a vital role in the quality and quantity of crop production. Thus, effective scientific methods for preserving soil quality are crucial to guaranteeing worldwide food security. Soil quality is monitored by a wide range of methods, including qualitative, semi-quantitative, and quantitative methods based on field and laboratory analyses. One of the most common quantitative approaches is to adopt soil quality indices (SQIs) [2,3]. An SQI can often be used as a measure of soil function, as it considers multiple soil properties to provide an overall assessment of soil health and productivity [4]. This is useful as individual soil property measurements may not be beneficial to farmers as they do not provide much information when out of context. Providing a complete assessment of the soil could help farmers to better develop proper management strategies for their crop production. Typically, for SQI analysis, a combination of soil biological, chemical, and physical properties is used, as this offers a more holistic evaluation of soil quality compared to analyzing individual parameters [5,6]. This is carried out by first creating a minimum data set (MDS) from a range of soil properties, which are then normalized into unitless scores, and then calculating the weighted sum of these properties [7]. A higher SQI score would indicate better soil function, as it means the soil has a greater capacity to perform. This approach can help farmers identify areas of concern and implement targeted interventions to improve soil health and increase crop yield.

A comprehensive assessment of soil quality involves measuring various soil parameters. However, this may be limited by the cost and complexity of the laboratory analyses required, which often require hazardous chemicals that may be destructive to the sample [8]. To address this challenge, an alternative approach is to utilize spectroscopic data collection, which can replace traditional laboratory analyses. Environmentally friendly techniques such as visible-near-infrared (vis-NIR) spectroscopy have enabled the rapid acquisition of soil data [9,10,11]. It is a cheap and non-destructive method in which multiple properties can be simultaneously predicted from a single spectrum [12]. Several studies have shown the accurate prediction of various soil properties such as soil organic matter (SOM), soil texture, and cation exchange capacity (CEC) [13]. In addition, several biological, chemical, and physical soil properties that have been suggested as useful and practical indicators for soil function assessment have also been predicted using vis-NIR spectroscopy, including pH, bulk density, and elemental concentrations [14,15,16]. The process typically begins by scanning all soil samples to obtain spectral data. The raw spectral data are then subjected to pre-processing to eliminate noise and unwanted variations. To build a reliable model, a calibration set, and a validation set are created. The calibration set is used to construct the model, while the validation set assesses its accuracy and reliability. Upon completing the calibration and validation steps, the model becomes capable of predicting soil properties for new soil samples using their vis-NIR spectral data.

This study looked at three different models, including Partial Least Squares Regression (PLSR), Random Forest (RF), and Cubist. PLSR is one of the most widely used methods for regression analysis due to its ease of interpretation, computational efficiency, and ability to handle large data sets [17]. On the other hand, Cubist is a rule-based machine learning framework that combines decision trees and linear regression for modelling [18]. Due to its ability to handle nonlinear relationships and interactions, it is able to capture complex patterns based on spectral data. RF is another commonly used machine learning technique for classification and regression problems that is known for its robustness and resistance to overfitting [19].

Soil fertility is considered an important soil function for sustainable agriculture and healthy ecosystems. The decline in soil fertility is an issue in many regions worldwide and a persistent limitation to agricultural productivity. Soil fertility is primarily associated with soil nutrient availability, pH, and organic matter (OM) [20]. For this study, the selected soil properties for the SQI are pH, OM, Potassium (K), Calcium (Ca) and Magnesium (Mg), available Phosphorous (P), and total Nitrogen (TN). N, P, and K are essential for the growth and development of plants and crops. Ca and Mg help to increase the availability of essential nutrients in the soil, maintain pH, improve soil structure, and make it more fertile. OM and pH are important soil quality indicators as they affect many soil functions and have a key role in fertility and nutrient availability [2].

Significant advancements have been made in the estimation of SQI for different types of soils and management practices [4,21,22]. However, only a few studies have used the vis-NIR spectrometer approach for SQI assessment. For example, Askari et al. [7] explored the capabilities of vis-NIR spectroscopy in predicting SQI related to soil productivity. The results indicated that SQI can be accurately predicted in both grassland (R^2^ = 0.92) and arable (R^2^ = 0.89) management systems, highlighting the effectiveness of vis-NIR spectroscopy in soil monitoring. Similarly, Veum et al. [23] and Paz-Kagan et al. [4] also used vis-NIR spectroscopy to calculate SQIs from predicted soil properties. However, these studies primarily focused on the top layers of soil and neglected the vertical distribution of SQIs throughout the soil profile. Soil properties and characteristics can vary significantly with depth, and neglecting the vertical distribution of SQIs may result in an incomplete understanding of the soil’s health and productivity. Additionally, most of these studies focused on a single method for calculating the SQI, either using lab-measured soil properties [24] or spectrally derived soil properties [3]. A limitation of these studies is their sample size, where 80 to 100 samples were used for SQI assessment. With a small sample size, the findings may not fully capture the variability present in the study area. In the study conducted by Gozukara et al. [3], a single soil profile was examined, measuring 1 m deep and 1 m wide. Soil samples were collected at 10 cm intervals along the profile. It is important to note that the findings of this study would be limited to this particular soil profile and location, so extrapolating the results to other regions or soil types would be difficult as soil characteristics and environmental conditions can vary significantly.

Calculating the SQI is a complex process, and there are multiple ways of calculating it; however, there is no comprehensive information on which approach is the best. There is a need to develop a robust, reliable, and user-friendly SQI that can be easily compared with existing methods. To achieve this, it is important to compare and evaluate various available methods for estimating SQI and identify the most suitable approach for a given soil and management system. This will enhance the accuracy and reliability of SQI estimation, leading to improved soil quality evaluation and management strategies.

The objective of this study is twofold. Firstly, to evaluate the effectiveness of vis-NIR spectroscopy in predicting soil properties utilizing a robust and diverse dataset with variable soil types. Secondly, to assess the efficacy of vis-NIR spectroscopy to predict SQI. By achieving these objectives, the study aims to contribute to the advancement of soil quality assessment methods using vis-NIR spectroscopy as a rapid, non-destructive, and cost-effective tool for predicting soil properties and estimating SQIs.

## 2. Materials and Methods

### 2.1. Study Soils and Study Area

**Figure 1:**
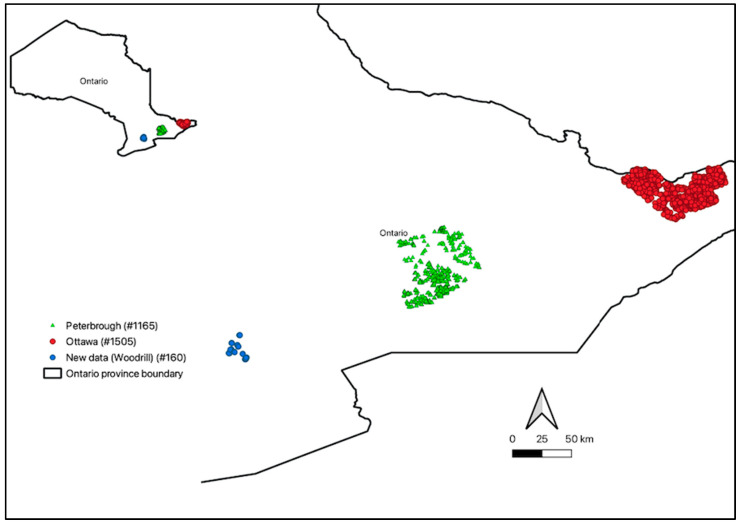
Map of Ontario showing the distribution of soil profile samples collected in Peterborough (green), Ottawa (red), and Woodrill (Dufferin and Wellington Counties) (blue). The numbers in brackets indicate the number of soil profiles collected from the area.

For this study, archived soil samples with laboratory data from three completed and ongoing soil survey projects will be analyzed. The soil samples were collected between the years 2016 and 2018 from various regions in Ontario. A total of 2830 soil profiles were collected, including 1165 soil profile samples from Peterborough, 1505 soil profile samples from Ottawa, and 160 soil profile samples from Dufferin and Wellington County (farms managed by Woodrill Limited, Guelph, ON, Canada) (Figure 1). This totaled up to 9461 soil horizon samples. These soil samples were collected from various land uses, including agriculture (37%), grasslands (19%), forests (25%), shrubs (11%), wetlands (5%), and others, which include uncultivated or unknown areas (3%).

The archived samples were air-dried, ground, and sieved to 2 mm. Soil properties such as pH, elemental concentrations (N, P, K, Mg, and Ca), and OM have previously been determined with traditional laboratory methods. Detailed laboratory methods for each soil property can be viewed in [25].

### 2.2. Spectral Collection

The ASD FieldSpec 4 spectrometer was utilized to conduct soil scanning, covering the visible spectrum (400–700 nm range) and the near-infrared spectrum (700–2500 nm range). Prior to scanning, calibration was performed on the spectrometer using a mylar wavelength reference. To ensure an accurate representation of the sample, each soil sample was tightly packed into a Petri dish and scanned three times in three different areas. The average of the three scans was considered for spectral analysis. At 10 min intervals, a blank wavelength was also collected to account for any potential interference or background noise.

### 2.3. Data Clean-Up and Preprocessing

In the processing of spectral data, data cleaning was performed at the initial stage to reduce noise and eliminate unnecessary information, thereby enhancing the data’s quality and accuracy. The R packages ‘prospectr’, ‘caret’, and ‘elmNN’ were utilized for all spectral cleaning and preprocessing transformations.

The spectral data with wavelengths below 399 nm and above 2451 nm were removed to prevent edge effects [25]. The soil’s chemical composition and structural attributes can cause non-linear light scattering, leading to non-linear relationships between absorption spectra and the desired measurement, which can affect measurement accuracy [26]. Furthermore, physical variables such as moisture, soil particle size, structure, and instrumental factors can also influence the quality of the spectra. To mitigate these effects, various preprocessing methods can be utilized, including Savitzky–Golay, 1st derivative, 2nd derivative, and gap derivative, to reduce baseline variation and increase spectral peak resolution [27]. Standard Normal Variate (SNV) can be applied to center and scale each individual spectrum, correcting for light scatter [26]. Additionally, the SNV-detrend technique can reduce curvature and eliminate wavelength-dependent scattering effects [28]. All the above preprocessing methods were tested, from which the SNV algorithm showed the best results; thus, it will be used for the current study for all predictions.

### 2.4. Modeling

Three different models were used for the prediction of various soil properties and SQI including, Partial Least Squares Regression (PLSR), Cubist, and Random Forest (RF).

During prediction analysis, a calibration set and validation set were created for each model to assess the performance of the model and avoid overfitting. The data were split ¾ calibration/validation and ¼ external validation using the Kennard–Stone algorithm with ‘prospectr’ and ‘multivariance’ packages in RStudio. The calibration/validation set was further split into a calibration set (¾) and a validation set (¼). The calibration set is used to build the model and determine the optimal parameters for the model. The validation set is used to evaluate the performance of the model by comparing the predicted values to the actual values. By using another separate validation set (an external validation set), the model can be tested on new, unseen data to ensure that it is generalizing well and not just memorizing the training data. This helps to prevent overfitting. The use of a calibration and validation set helps to ensure that the model is robust and has good predictive power.

### 2.5. SQI

**Figure 2:**
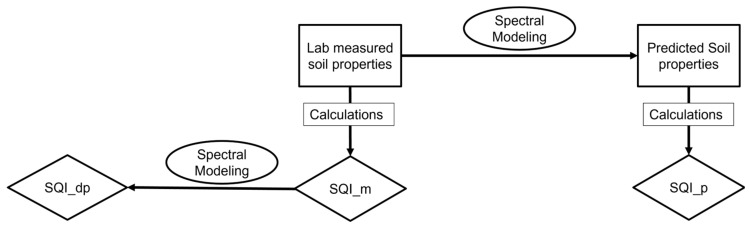
Flowchart of three different approaches used to examine the soil quality index including SQI_dp (direct prediction of soil quality index), SQI_m (measured soil quality index), and SQI_p (predicted soil quality index).

SQI is a metric that can be used to quantify the overall health and fertility of soil. For this study, three different approaches were utilized to estimate SQI, measured SQI (SQI_m), predicted SQI (SQI_p), and direct prediction of SQI (SQI_dp). Figure 2 includes a flowchart to easily visualize the three different approaches.

Measured SQI is calculated using traditional laboratory-measured soil properties such as OM, pH, electrical conductivity, and available nutrients. Although this method is frequently employed in soil analysis, it can be time-consuming and expensive. Predicted SQI is calculated in a similar way to SQI_m; however, instead of using laboratory-measured soil properties, this approach uses spectral data collected from vis-NIR spectroscopy to develop a model that can predict soil properties, which can then be used to calculate the SQI. This approach is faster and less expensive than the measured SQI approach. Lastly, SQI_dp is directly predicted using spectral data without the need to calculate individual soil indicators. This approach is the fastest and least expensive of the three, but it requires a large amount of high-quality spectral data and a robust machine learning model. SQI assessment involves three steps: (1) selecting the representative indicators from the full set of measured soil parameters to form the minimum data set (MDS); (2) transforming the MDS indicators into scores; and (3) integrating the scores to form the SQI [29].

### 2.6. Selecting the Minimum Data Set

The MDS can be selected using various methods such as principal component analysis (PCA), expert opinion (EO), and factor analysis [30]. For this study, we used EO by looking at the available literature and the knowledge of soil scientists. In this approach, primary soil properties were selected based on EOs with regard to their established role in soil fertility (soil properties listed in Table 1). OM and soil pH are critical indicators of soil quality as they significantly impact various soil functions. These two parameters play a crucial role in determining soil fertility and the availability of essential nutrients for plant growth and development [2]. Additionally, the availability of certain key nutrients such as N, P, and K are vital for the growth and development of plants. Ca and Mg also play an important role in soil fertility as they increase the availability of other essential nutrients, regulate soil pH, improve soil structure, and contribute to overall soil fertility [20,31].

### 2.7. Transformation of the MDS Indicators and Weight Assignment

The soil properties selected for the study have different units of measurement. To standardize them, they were transformed and normalized into a unitless score ranging from 0 to 1 using either linear or non-linear scoring methods. The indicators were scored based on their impact on soil fertility. If an indicator has a positive effect on soil fertility, it was labelled as “more is better”, whereas if it has a negative effect, it was given a “less is better” score and if the indicator has both positive and negative effects, it was assigned an “optimum” [24]. For this study, the non-linear equation was utilized:

(1) $$\mathrm{Si}=\frac{1}{\left(1+{\left(\frac{x}{x_{o}}\right)}^{-/+b}\right)}$$

where Si is the non-linear score, *x* is the value of the selected indicator, *x_o_* is the mean value of each indicator, and *b* is the slope, which was set as −2.5 for “more is better” and 2.5 for “less is better” functions [24]. The non-linear equation is preferred over the linear method because it has been shown to determine the function better, thus it will provide a more comprehensive and realistic assessment of soil fertility [22,24]. The choice of −2.5 and 2.5 is based on previous studies. The value 2.5 is used for the slope because it has been found to provide a good fit for the non-linear relationship between soil quality and several soil properties. For example, Yu et al. [32] found that the non-linear equation with the slope as 2.5 provided a good fit for the relationship between soil quality and several soil properties, including pH, TN, soil organic content, and P.

In this study OM, N, P, K, Ca, and Mg were scored as “more is better” and pH was scored as “optimum”. In the case for pH, the “more is better” function was considered up to a threshold range (pH of 5.5–7.0), Munroe [33], after which the “less is better” function was used to generate the scores.

Weights were assigned based on EOs and the literature. Although the PCA method is a common approach for calculating weights [22], it was not utilized in this study. This decision was made due to the presence of missing data, which can adversely affect the accuracy and reliability of the PCA results. Therefore, alternative approaches were employed to address the limitations associated with missing data and ensure the validity of the weight assignment process.

The allocation of weights in this study was based on the significance of soil indicators in relation to soil fertility. Greater weight was assigned to the indicators that exerted the most substantial influence on soil fertility. Weights were then further divided among the indicator parameters based on the relative importance of each indicator (Table 1). A similar approach was used by [34]. OM is a key component of soil fertility, as it provides nutrients, enhances soil structure, and promotes soil microbial activity, thus OM is assigned a high weight in this soil fertility assessment. Soil pH affects the availability of nutrients (N, P, K) to plants and can also influence soil microbial activity, thus is also given a higher weight. N is a critical nutrient for plant growth and is often a limiting factor in agricultural systems, which is why it was given a higher weight compared to P and K. Ca and Mg are also important nutrients for plant growth and soil structure; however, they are usually present in adequate quantities, thus were assigned lower weights.

The weighted MDS indicator scores for each observation were summed up using the following equation:

(2) $$\mathrm{SQI}=\sum_{i=1}^{i=n}Wi\times Si$$

where *Wi* is the weight assigned to each selected indicator and *Si* is the score of each indicator. As discussed above, three approaches to SQI will be assessed, including: (1) measured SQI, which is derived from laboratory-measured soil properties; (2) predicted SQI, which is calculated using predicted soil properties from spectral data; and (3) direct measurement of SQI, which is determined using PLSR, Cubist, and RF algorithms based on the measured SQI.

The evaluation of the model was carried out by the Coefficient of Determination (R^2^). The evaluation of each model and comparison of the three SQIs were based on descriptive statistics, scatter plots, and R^2^ values. Statistical analyses were performed using the RStudio programming language.

## 3. Results

### 3.1. Descriptive Statistics of Measured Soil Properties

The soil properties were obtained using previous measurement procedures, with the specific sampling methods used for each soil property documented in detail in the publication by Vestergaard et al. [25] The results of the laboratory measurements of the soil properties are summarized in Table 2. The mean, median, minimum, maximum, and standard deviation for each soil property is presented in the table.

The analysis of laboratory-measured OM at the study site revealed a mean value of 5.33%, with a median of 1.78%. The minimum and maximum values observed were 0 and 85.17%, respectively. The high standard deviation of 12.50 suggests a high degree of variability in OM content. Similar calculations were performed for pH, TN, P, K, Ca, and Mg.

P, K, Ca, and Mg all had high standard deviations, which can be attributed to the large size of the study site. The study site encompasses a variety of soil types and land uses, each with its own unique characteristics, leading to a high degree of variability in soil properties. In addition, differences in topography, vegetation, and other factors within the study site can also contribute to the variability in soil properties [35], as evidenced by the high standard deviations and the minimum and maximum values.

Soil properties were estimated using vis-NIR spectroscopy with PLSR, Cubist, and RF. The performance of each model is looked at through the calibration, validation, and external validation data sets. The R^2^ values of the calibration set indicate the level of fit between the model and its parameters. The R^2^ values for the calibration set in PLSR, Cubist, and RF models range from 0.29 to 0.81, 0.85 to 0.94, and 0.85 to 0.97, respectively (Table 2). A high R^2^ value for the calibration set indicates a good fit of the model to the data; however, it may also indicate overfitting, where the model is too complex and instead memorizing the training data, thus not generalizing well to new data [36].

The validation R^2^ values provide a useful insight into the generalization ability of the models by giving an estimate of their performance on unseen data. The external validation R^2^ values, obtained by using an independent data set, offer a more accurate prediction of the model’s performance since they are tested on new and untested data. By using these R^2^ values, we can gain a deeper understanding of the effectiveness of the model.

Table 3 compares the validation and external validation R^2^ values for PLSR, Cubist, and RF models for the selected soil properties. The R^2^ values for the validation of PLSR, Cubist, and RF ranged from 0.36 to 0.84, 0.45 to 0.92, and 0.27 to 0.89, respectively, whereas, in the external validation, the R^2^ values for PLSR, Cubist, and RF ranged from 0.26 to 0.82, 0.35 to 0.93, and −0.044 to 0.90, respectively. The results show that the Cubist model performed best in predicting all selected soil properties (OM, pH, N, P, K, Ca, and Mg) based on having the highest R^2^ values. PLSR performed second best, and RF underperformed for most soil properties except for OM and TN. Similar results were seen in Zhang et al. [37] and Dangal et al. [18] where Cubist overperformed in predicting similar soil properties compared to PLSR and RF (R^2^ > 0.75). Furthermore, when looking at the calibration set, the R^2^ always seems to be higher for each soil property, especially in the case of RF, indicating an overfitting situation. Overfitting usually occurs when the model fits the calibration data too well, capturing noise or random variations in the data that do not generalize to new, unseen data, hence giving lower R^2^ values in the external validation set [36].

### 3.2. Measured SQI, Predicted SQI and Direct Prediction of SQI

Three methods for evaluating SQI were employed to assess soil fertility. SQI_m was calculated using laboratory-measured soil properties. The predicted soil properties from the Cubist model were used for the calculation of predicted SQI due to their high R^2^ values (Table 3). SQI_dp, on the other hand, was estimated by utilizing a combination of measured SQI, soil property data, and spectral data using PLSR, Cubist, and RF. This approach provides an alternative method for evaluating the soil quality, allowing for a more comprehensive understanding of the soil’s fertility.

**Figure 3:**
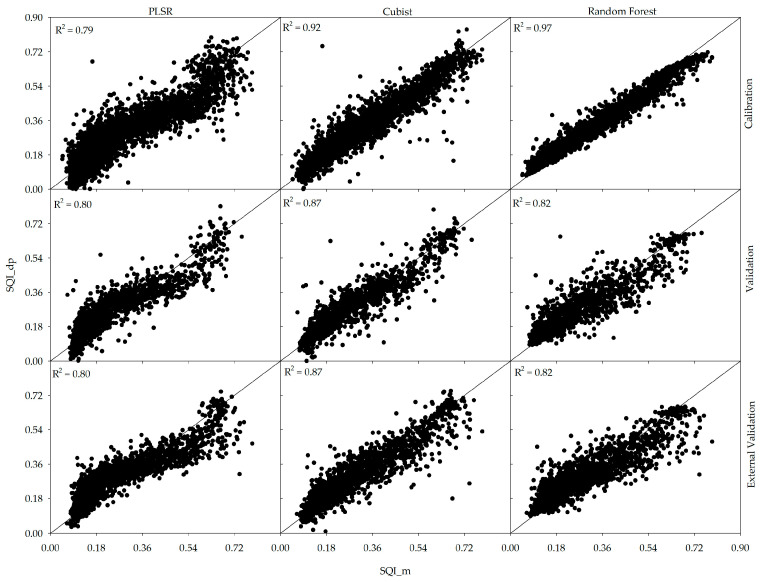
Scatterplots of measured versus direct prediction of soil quality index (SQI) using three different models (PLSR, cubist, and RF) for the calibration, validation, and external validation datasets.

Based on a visual examination of Figure 3, the RF and PLSR models show more variability or scatter in the data than Cubist when considering the validation and external validation indices. All three models gave good R^2^ values; however, Cubist appeared to be the best performing model and most effective in directly predicting SQI (R^2^ = 0.87 based on validation and external validation). Therefore, for the purpose of comparison, the SQI predicted by the Cubist model will be evaluated against the measured and predicted SQI values.

The average results for each SQI method (measured, predicted, and direct prediction) were found to be closely aligned for SQI_m and SQI_dp, suggesting that both methods capture similar information regarding soil quality (Table 4). SQI_m ranged from 0.047 (very low soil quality) to 0.79 (good soil quality). On the other hand, the SQI_p and SQI_dp values ranged from 0.069 to 0.76 and 0 to 0.84, respectively. These wide ranges in SQI values can be attributed to the inclusion of different land uses in the analysis. Agricultural lands generally possess higher soil fertility compared to non-agricultural lands. However, it is worth noting that for the majority of samples, the SQI values fell within the range of 0.25 to 0.50, indicating low to moderate soil quality. The wide range of SQI values highlights the variability in soil quality across different land uses.

**Figure 4:**
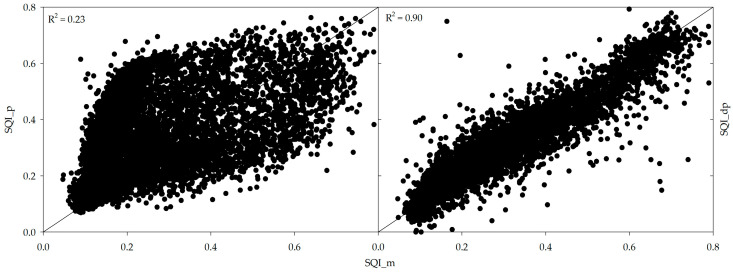
Scatter plots of measured soil quality index (SQI_m) against SQI_p (predicted soil quality index) and SQI_dp (direct prediction of SQI) using Cubist model.

Plotting SQI_m on the x-axis and SQI_p and SQI_dp on the y-axis, it can be seen that SQI_m is more closely related to SQI_dp values compared to SQI_p, which can be visualized by the high variability in data between measured versus predicted SQI graph (Figure 4). The R^2^ for predicted SQI and direct prediction of SQI are 0.23 and 0.90, respectively. The poorer performance of SQI_p compared to that of SQI_dp was possibly due to the accumulation of prediction errors. When predicting SQI using predicted soil properties, there is a potential for error accumulation at each stage of prediction. Errors in estimating individual soil properties can propagate and accumulate, leading to a less accurate overall prediction of SQI. In contrast, SQI_dp from spectral data bypasses the intermediate step of predicting soil properties, thereby reducing the potential for error propagation.

## 4. Discussion

### 4.1. Soil Property Prediction

Spectroscopy in the visible and near-infrared spectral ranges can be utilized to assess SQI and evaluate soil fertility by measuring various soil properties. In this study, the Cubist model effectively predicted all soil fertility indicators (OM, pH, TN, P, K, Ca, and Mg). OM and TN were predicted well overall compared to the other soil properties. This is because, in the vis-NIR regions, these properties have direct interactions with specific absorbance bonds [10]. The use of spectral analysis and machine learning algorithms (PLSR, Cubist, and RF) is rapid, making it possible to analyze a large number of samples in a practical and timely manner as a prediction model. The use of these algorithms enables quick and effective predictions (R^2^ values > 0.50 for most soil properties), making it a practical solution for many applications in the field. These advantages make spectral analysis in combination with PLSR, Cubist, and RF algorithms an attractive method for environmental monitoring, especially for modeling SQIs.

### 4.2. Measured SQI, Predicted SQI and Direct Prediction of SQI

Measured SQI is the most accurate representation of soil quality, as it is based on direct measurements of soil properties; however, it has its limitations. A few being that it is time-consuming, laborious, and expensive to measure each soil property. SQI_p involves predicting individual soil properties, which are then used as indirect indicators of SQI. This approach is faster; however, it was found to be inaccurate due to the error propagation issues. It was observed that the prediction of certain soil properties, such as Mg (R^2^ = 0.50), K (R^2^ = 0.48), and available P (R^2^ = 0.35), were not as accurate as that for other properties. As a result, there were discrepancies between SQI_p and SQI_m (Figure 4). On the other hand, spectrally derived SQI uses the spectral data to directly predict the SQI and bypass the error propagation issues. Furthermore, this approach has its benefits, which include a reduction in sampling time and an increase in the number of samples that can be analyzed within time and budget constraints. It is a rapid, non-destructive, reproducible, and cost-effective analytical method and, therefore, a promising tool for soil quality assessment. In the current study, it was observed that measured and direct prediction SQIs are more closely related compared to measured and predicted SQIs as illustrated in Figure 4. This indicates that the direct prediction approach using vis-NIR spectroscopy shows promise in providing reliable assessments of soil quality.

### 4.3. SQI and Soil Fertility

This study utilized a combination of EOs and existing literature to identify the soil indicators that best reflect soil fertility. Other studies, such as those conducted by Lenka et al. [34] and Vasu et al. [30], also utilized EOs to determine soil indicators. Both Vasu et al. [30] and Lenka et al. [34] compared the effectiveness of the PCA method and the EO method in determining soil indicators and found that the EO approach resulted in a higher correlation with the SQI. They noted that the higher correlation with the EO approach was likely due to the inclusion of representative variables in the MDS, which more accurately captured the specific factors being considered for their research [34]. Vasu et al. [30] also found that SQI computed by the EO method to be better correlated with crop yield than the PCA method.

For this study, OM, pH, N, P, K, Ca, and Mg were used as soil indicators to represent soil fertility. Soil OM is an important indicator of soil fertility, as it provides essential nutrients and energy to soil microorganisms, which in turn, support plant growth [38]. The pH of soil can impact the availability of essential nutrients to plants, as some nutrients are more readily available at certain pH levels. Soil nutrients, such as N, P, and K, are essential for plant growth and can be used to assess soil fertility [39]. Furthermore, Ca and Mg are also important micronutrients that can impact soil fertility, as Ca plays a crucial role in maintaining pH, which can impact the availability of other nutrients. Mg affects the production of plants and crops as it is an essential component of the chlorophyll molecule involved in photosynthesis [40]. A high SQI value would indicate that the soil is fertile and healthy, while a low SQI value would indicate that the soil is less fertile and may require improvement.

To date, limited research has been conducted on the use of spectroscopy for the direct assessment of SQI through the evaluation of soil properties related to soil health and fertility. Existing studies have primarily focused on using predicted individual soil indicators to determine soil quality for a specific soil type or land use [4,6,29,41]. In a study conducted by Askari et al. [7], three different SQIs were compared (measured, predicted, and direct prediction) in grassland and airable land. The results showed that the prediction of SQI using spectral measurements resulted in an excellent correlation (R^2^ = 0.89) and was significantly different from the predicted SQI (*p* < 0.05). However, there was no significant difference found between the spectrally derived SQI and the measured SQI [7]. A similar conclusion was reached in Yang et al. [42] where the Soil Fertility Index (SFI) estimated directly from vis-NIR spectra was superior (R^2^ = 0.92) to the SFI calculated from predicted individual indicators (R^2^ = 0.84). This study also found no significant difference between the measured SQI and the direct prediction of SQI. A limitation of Askari et al. [7] and Yang et al. [42] is their use of only the top layer of soils for SQI assessment. It is generally better to calculate SQI from the whole soil profile, as it provides a more accurate indication of the overall soil fertility. The top layer of the soil can be impacted by different factors, such as weather, which may not be applicable to the rest of the soil profile. Additionally, when assessing soil fertility using SQIs, the deeper layers of the soil may contain nutrients and other elements that contribute to overall fertility but may not be as evident in the top layer [3]. By taking the whole soil profile into account, we can obtain a more accurate picture of SQI and soil fertility. The sample size is also very low in these studies; having a larger sample size enhances the robustness of the results in SQI analysis. More samples increase the representativeness of the dataset and reduce uncertainty. Furthermore, with a larger sample size, the findings are more likely to be reliable, generalizable, and meaningful.

The present study represents a significant advancement in soil quality assessment, distinguishing itself through the innovative use of visible near-infrared (vis-NIR) spectroscopy. This approach addresses a critical gap in traditional soil quality analysis—its labor-intensive and costly nature. By harnessing the Cubist model, our study achieved impressive accuracy levels (R^2^ = 0.35–0.93) in predicting essential soil indicators. This outcome underscores the superiority of our method over conventional laboratory-based analyses, both in terms of efficiency and environmental friendliness. The use of vis-NIR spectroscopy coupled with the Cubist model marks a substantial leap forward in soil science. This technology simplifies the process of measuring critical soil indicators like pH, OM, K, Ca, Mg, P, and TN. Our study successfully demonstrated that SQI can be directly predicted with a high accuracy (R^2^ = 0.90) using vis-NIR spectroscopy. This direct prediction method (SQI_dp) outperformed the predicted SQI (SQI_p) calculated from spectral data-derived soil properties. By integrating multiple soil properties, our approach provides a more holistic assessment of soil health compared to traditional methods, which often focus on individual parameters.

Farmers can utilize the findings of this study to understand soil fertility more comprehensively, allowing for informed decision-making in crop management and farming practices. The integration of vis-NIR spectroscopy into soil analysis represents a significant contribution to sensor technology, enabling rapid and non-destructive soil testing. This study offers a valuable tool for managing natural resources more sustainably, particularly in the context of soil conservation and land use planning. The precision and efficiency of vis-NIR spectroscopy align perfectly with the goals of precision agriculture, enabling farmers to optimize inputs and enhance crop yields while minimizing environmental impact. Our approach provides a rapid, cost-effective method for ongoing soil health monitoring, critical for maintaining soil quality over time.

The methodology developed in this study has far-reaching implications, not just for agriculture but for broader environmental and ecological management. The ability to quickly and accurately assess soil quality is paramount in the era of climate change and environmental degradation. This study’s implications extend to areas such as sustainable farming practices, the mitigation of soil degradation, and the enhancement of crop productivity, all of which are critical in the global effort to ensure food security and environmental sustainability. Additionally, the application of this research can be extended to other fields where soil quality is a key factor, such as in the restoration of degraded lands, urban planning, and even in the assessment of the impact of various land use practices on soil health.

In summary, this study not only advances the field of soil science but also contributes significantly to the realms of precision agriculture, sensor technology, and natural resource management. The practical applications of this research are vast, offering new avenues for enhancing soil health and agricultural productivity. The adoption of this innovative approach could lead to more sustainable agricultural practices worldwide, ultimately contributing to global food security and environmental conservation.

## 5. Conclusions

This study underscores the integral role of soil quality indices (SQIs) in advancing agricultural practices and crop management. SQIs emerge as a vital tool for farmers, offering insights into the fertility, productivity, and sustainability of their soils. This knowledge is pivotal in optimizing agricultural methodologies to enhance crop yields, improve soil health, and foster environmental stewardship. Furthermore, the early identification of soil-related issues such as degradation, nutrient imbalances, and erosion risks, facilitated by SQIs, is crucial for timely and effective soil management interventions [5].

Our findings highlight the nuanced relationship between SQIs and land use. The study reveals that SQIs are not universal but are instead intricately linked to the specific land use of a site. This is a critical consideration, as different land uses—ranging from agriculture to urbanization, forestry, and mining—impart unique impacts on soil’s physical, chemical, and biological properties. A notable limitation of our approach was the amalgamation of samples from diverse land uses in the SQI analysis. Future research could refine this understanding by categorizing samples based on land use before conducting SQI evaluations, thereby elucidating the distinct effects of various land uses on soil quality.

Additionally, our study’s reliance on laboratory-based SQI analysis, while beneficial for ensuring accuracy and consistency, may not fully capture the complexities of field conditions. External factors such as soil moisture fluctuations and disturbances like soil roughness or vegetation cover, which significantly influence soil properties, are often overlooked in controlled laboratory environments. Therefore, we advocate for the development of field-applicable spectroscopic methods for soil quality assessment, which would offer a more realistic and comprehensive evaluation of soil health, guiding informed soil management practices.

In the realm of soil property prediction, our research demonstrates the efficacy of visible and near-infrared (vis-NIR) spectroscopy. The Cubist model, in particular, exhibits superior performance over Partial Least Squares Regression (PLSR) and Random Forest (RF) in predicting individual soil properties. The correlation coefficients (R^2^) for key soil indicators—organic matter (OM), nitrogen (N), pH, calcium (Ca), magnesium (Mg), potassium (K), and phosphorus (P)—are notably high, indicating the robustness of this approach. Moreover, the direct prediction of SQI using vis-NIR spectroscopy with the Cubist model is shown to be highly accurate. The model-derived SQI (SQI_m) aligns more closely with depth-profiled SQI (SQI_dp, R^2^ = 0.90) than with predicted SQI (SQI_p, R^2^ = 0.23), suggesting that vis-NIR spectroscopy is a potent tool for directly assessing soil functions, particularly fertility.

In conclusion, this study contributes significantly to the field of soil science by providing a nuanced understanding of the relationship between soil quality indices and land use, and by demonstrating the potential of vis-NIR spectroscopy in soil quality assessment. These insights are invaluable for advancing sustainable agricultural practices and enhancing soil health, with profound implications for environmental conservation and food security.

## Figures and Tables

**Table 1:** Weight assignment for each soil indicator and its scoring function.

Indicators	Weights	Scoring Function
OM %	0.35	More is better
pH_H_2_O	0.20	Optimum
TN %	0.15	More is better
P μg/g	0.10	More is better
K mg/L soil dry	0.10	More is better
Ca mg/L soil dry	0.05	More is better
Mg mg/L soil dry	0.05	More is better

**Table 2:** Descriptive statistics of measured soil properties where s is standard deviation, n is number of samples.

Soil Property	Mean	Median	Min	Max	s	n
OM %	5.33	1.78	0.00	85.17	12.50	9452
pH_H_2_O	6.95	7.16	3.29	9.07	0.97	9459
TN %	0.24	0.11	0.00	3.30	0.37	8789
AvailP μg/g	38.34	5.75	0.26	1506.00	1.35	8825
K mg/L dsoil	200.54	80.95	1.90	6688.00	465.25	8888
Ca mg/L dsoil	4235.08	2940.00	18.20	157,040.00	9944.54	9158
Mg mg/L dsoil	316.18	187.00	8.20	4240.00	333.87	8722

**Table 3:** Statistical data on soil properties predicted by PLSR, Cubist, and RF models with their respective R^2^ values.

Soil Properties	Calibration			Validation			External Validation		
	PLSR	Cubist	RF	PLSR	Cubist	RF	PLSR	Cubist	RF
OM %	0.80	**0.94**	0.97	0.84	**0.92**	0.89	0.81	**0.93**	0.90
pH_H_2_O	0.65	**0.87**	0.93	0.66	**0.83**	0.54	0.36	**0.70**	−0.044
TN %	0.78	**0.94**	0.97	0.77	**0.87**	0.84	0.82	**0.92**	0.90
Avail P μg/g	0.31	**0.87**	0.88	0.36	**0.45**	0.27	0.26	**0.35**	0.16
K mg/L soil	0.28	**0.85**	0.85	0.42	**0.53**	0.34	0.31	**0.48**	0.19
Ca mg/L soil	0.50	**0.92**	0.87	0.41	**0.69**	0.48	0.49	**0.63**	0.41
Mg mg/L soil	0.74	**0.86**	0.94	0.54	**0.67**	0.45	0.31	**0.50**	0.28

Note: The bold-faced numbers show the highest performance models.

**Table 4:** Descriptive statistics of measured SQI, predicted SQI and direct prediction of SQI where *s* is the standard deviation and *n* is the number of samples.

	Mean	Median	Min	Max	*s*	*n*
Measured SQI	0.27	0.22	0.047	0.79	0.16	8093
Predicted SQI	0.35	0.34	0.069	0.76	0.17	8093
Direct prediction of SQI	0.27	0.23	0.000	0.84	0.15	8093

## Data Availability

Authors do not have permission or authority to make the data available publicly.
